# The Age in Swimming of Champions in World Championships (1994–2013) and Olympic Games (1992–2012): A Cross-Sectional Data Analysis

**DOI:** 10.3390/sports4010017

**Published:** 2016-03-04

**Authors:** Beat Knechtle, Nicola Luigi Bragazzi, Stefan König, Pantelis Theodoros Nikolaidis, Stefanie Wild, Thomas Rosemann, Christoph Alexander Rüst

**Affiliations:** 1 Gesundheitszentrum St. Gallen, St. Gallen 9001, Switzerland; 2 Department of Health Sciences (DISSAL), School of Public Health, University of Genoa, Genoa 16132, Italy; robertobragazzi@gmail.com; 3 Department of Neuroscience, Rehabilitation, Ophthalmology, Genetics, Maternal and Child Health (DINOGMI), University of Genoa, Genoa 16132, Italy; 4 Institute of Primary Care, University of Zurich, Zurich 8091, Switzerland; steven.king@gmx.ch (S.K.); stefanie.wild@gmx.ch (S.W.); thomas.rosemann@usz.ch (T.R.); christoph.ruest@bluewin.ch (C.A.R.); 5 Department of Physical and Cultural Education, Hellenic Army Academy, Vari 16673, Greece; pantelisn@phed.uoa.gr

**Keywords:** champions, age, performance, sex difference

## Abstract

(1) Background: We investigated the age of swimming champions in all strokes and race distances in World Championships (1994–2013) and Olympic Games (1992–2012); (2) Methods: Changes in age and swimming performance across calendar years for 412 Olympic and world champions were analysed using linear, non-linear, multi-level regression analyses and MultiLayer Perceptron (MLP); (3) Results: The age of peak swimming performance remained stable in most of all race distances for world champions and in all race distances for Olympic champions. Longer (*i.e.*, 200 m and more) race distances were completed by younger (~20 years old for women and ~22 years old for men) champions than shorter (*i.e.*, 50 m and 100 m) race distances (~22 years old for women and ~24 years old for men). There was a sex difference in the age of champions of ~2 years with a mean age of ~21 and ~23 years for women and men, respectively. Swimming performance improved in most race distances for world and Olympic champions with a larger trend of increase in Olympic champions; (4) Conclusion: Swimmers at younger ages (<20 years) may benefit from training and competing in longer race distances (*i.e.*, 200 m and longer) before they change to shorter distances (*i.e.*, 50 m and 100 m) when they become older (>22 years).

## 1. Introduction

Twenty-six years ago, Schulz and Curnow investigated the age of Olympic gold medal winners in swimming, competing between 1896 and 1980 [1]. The average age in freestyle swimming was ~18 years for women and ~21 years for men [1]. This age remained stable for almost a century [1]. Nevertheless, over time humans have undergone changes in body dimensions such as body height, body mass and slenderness, and swimming performance has improved continuously across the years [2,3,4].

However, there is no recent study investigating the age of today’s champions and the actual change of the age of peak swimming speed over time. In recent years, new swimming records were set continuously with a peak of new records at the World Championship in 2008 due to newly introduced swimsuits [5]. The full body swimsuits were banned worldwide by 1 January 2010. Considering long course championships, the fastest performance for women and men was obtained in 50 m freestyle at the World Championship in 2009. Britta Steffen finished in 23.73 s and Cesar Cielo in 21.08 s. The most recent world records were set in 2013 for women by Katie Ledecky [6,7] in the 800 m and 1500 m freestyle at age 16, by Ruta Meilutyte [8] in the 100 m breaststroke at age 16, and by Yuliya Efimova [9,10] in the 200 m breaststroke at age 21. For men, the latest world records were set at the World Championships in 2009. In total, 11 new records were achieved in that year for men, held in different strokes by Cesar Cielo [11,12] in the 50 m and 100 m freestyle at the age of 22; Paul Biedermann [13] in the 200 m freestyle at the age of 23; Lin Zhang [14] in the 800 m freestyle at the age of 22; Brenton Rickard [15] in the 100 m breaststroke at the age of 26; Daniel Gyurta [16] in the 200 m breaststroke at the age of 20; Junya Koga [17] in the 100 m backstroke at the age of 22; Aaron Peirsol [18] in the 200 m backstroke at the age of 18; Michael Phelps [19,20] in the 100 m and 200 m butterfly at the age of 24; and Ryan Lochte [21] in the 200 m individual medley at the age of 25. No new world record was set in 2013. Regarding the Olympic Games, the most recent Olympic records were set in 2012 for women by Ranomi Kromowidjojo in the 100 m freestyle at the age of 22; Allison Schmitt [22] in the 200 m freestyle at the age of 22; Camille Muffat [23] in the 400 m freestyle at the age of 23; Melissa Franklin [24,25] in the 100 m and 200 m backstroke at the age of 17; Dana Vollmer [26,27] in the 100 m butterfly at the age of 25; and Shiwen Ye [28,29] in the 200 m and 400 m individual medley at the age of 16. For men, the latest Olympic records were set in 2012 by Yang Sun [30] in the 400 m, 800 m, and 1,500 m freestyle at the age of 21; Cameron van der Burgh [31,32] in the 100 m breaststroke at the age of 24; by Metthew Grevers [33,34] in the 100 m backstroke at the age of 27; and by Michael Phelps [35] in the 200 m individual medley at the age of 27.

This last exceptional talent has four other unbroken Olympic records from 2008 in the 200 m freestyle [36], the 100 m [37] and 200 m [38] butterfly and the 400 m individual medley [39,40] set at the age of 23. Michael Phelps (USA) won 18 Olympic gold medals, two silver medals and two bronze medals [41]. He retired after the Olympic Games in London 2012 [42]. Nevertheless, Michael Phelps returned to consistent training in November 2013 [43,44]. In 2014, he won three gold and two silver medals at the Pan Pacific Championships in August [45]. Michael Phelps intends to compete in the Olympic Games in Rio de Janeiro in 2016 [46]. That would not be the first comeback in swimming history. Ian Thorpe (AUS) retired in 2006 at the age of 23 years after he won nine Olympic medals—a gold medal in the 400 m freestyle, in the 4 × 100 m and 4 × 200 m freestyle relay in 2000 at the age of 17, a silver medal in the 200 m freestyle, and in the 4 × 100 m medley relay in 2000 at 17 years of age, a gold medal in the freestyle 200 m and 400 m, a silver medal in the freestyle relay 4 × 200 m, and a bronze medal in the 100 m freestyle in 2004 at age 21 [47]. He made his first attempt at a comeback at the Olympic Games in London in 2012 at the age of 30, but failed. Ian Thorpe won nine Olympic medals (five gold medals, three silver medals, and one bronze medal) and 13 World Championship medals (11 gold medals, one silver medal, and one bronze medal) [48]. Recently, Thorpe talked about competing in the 2016 Rio Olympics [49]. In April 2014, he suffered from a serious infection after shoulder surgery [49], but he still hopes for a comeback [50]. Another swimmer who tried to make a comeback in swimming in earlier years was Mark Spitz (USA). His aim was a comeback at the Olympic Games in 1992 at over 42 years old—20 years after his last competition at the Olympic Games in Munich in 1972 [51]. Mark Spitz won in total nine gold medals, one silver medal, and one bronze medal at Olympic Games [52]. After Michael Phelps, who won eight gold medals in one competition at the Olympic Games in 2008 (Beijing), Mark Spitz is next with seven medals won at the Olympic Games in Munich in 1972. He failed in his comeback at the Olympic Games in 1992, being two seconds slower than the required qualifying time [53]. The Olympic champion Ed Moses, a member in the 4 × 100 m medley relay and silver medal winner in the breaststroke 100 m in 2000, wanted to join the Olympic team at the age of 32 in 2012, but failed [54,55].

These data show that records at World Championships and Olympic Games were achieved in athletes age 25 and younger. Michael Phelps (born 1985) plans his comeback at the age of 31 at the Olympic Games in 2016. The question may be raised regarding whether he will be able to perform in such a way as to win a medal at that age. For example, Dara Torres (born 1967) showed that it is possible to win a medal in a comeback at the Olympic Games. At the age of 14, Torres set her first world record. At the age of 17 she was Olympic champion in her hometown of Los Angeles in the 1984 Olympic Games in the 4 × 100 m freestyle relay. At the age of 25, she left sport, only to participate seven years later in the 2000 Olympic Games in Sydney and win five more medals. In April 2006, she gave birth to her daughter Tessa. At the 2008 Olympic Games in Beijing, at age 41, Dara Torres won three silver medals (4 × 100 m freestyle relay, 50 m freestyle, and 4 × 100 m medley relay) [56]. Inspired by the recent decisions of different swimmers to make comebacks at a competitive level, with this study we aimed to systematically investigate the age of winners of Olympic Games (1992 to 2012) and World Championships (1994 to 2013) for all race distances and strokes.

## 2. Materials and Methods

### 2.1. Ethics Approval

All procedures used in the study were approved by the Institutional Review Board of Kanton St. Gallen, Switzerland, with a waiver of the requirement for informed consent of the participants given the fact that the study involved the analysis of publicly available data.

### 2.2. Data Sampling and Data Analysis

All data for this study were obtained from www.swimrankings.net. This web page records all swimming race results according to events such as World Championships and Olympic Games. During data sampling and preparation for analysis, we found that data before 1992 were not complete for all strokes and distances, and we therefore started data analysis from the year 1992 onward. Full data were available going back to 1992 for 202 female and 210 male champions (*i.e.*, 412 swimmers in total), 250 in the World Championships and 162 in the Olympic Games. Prior to 2001, the 1500 m freestyle was not raced by women in the World Championships. Therefore, we have a difference of two swimmers in the number of female and male athletes. In the Olympic Games, there was a difference of six swimmers in the number of athletes between both sexes due to the missing races in the 1500 m freestyle during the whole period. All race distances which are held both in the World Championships (1994–2013) and the Olympic Games (1992–2012) were included. We excluded the 50 m breaststroke, the 50 m backstroke, and the 50 m butterfly, which were only held in the World Championships. Therefore, the following race distances were included and analysed: freestyle (50 m, 100 m, 200 m, 400 m, 800 m, and 1500 m), breaststroke (100 m, 200 m), backstroke (100 m, 200 m), butterfly (100 m, 200 m), and individual medley (200 m, 400 m). We analyzed all listed strokes and distances in both sexes and both in the World Championships and the Olympic Games for changes in the age and swimming performance of all champions over the years. Race times were converted to swimming speed using the following equation: (swimming speed (m·s^−1^)) = (race distance (m))/(race time (s)). The age of the athletes was calculated from the date of birth given at the web page from www.swimrankings.net.

### 2.3. Statistical Analysis

Descriptive values were computed as mean ± standard deviation (SD) for continuous variables. Normal distribution was tested using a D’Agostino and Pearson *omnibus* normality test and homogeneity of variances was tested using a Levene’s test. A MultiLayer Perceptron (MLP), which is a feed-forward Artificial Neural Network (ANN), with the hyperbolic tangent as activation function in the input/hidden layers, and the softmax activation function in the output layer and cross-entropy as error function, was used to assess the impact of each co-variable on the swimming performance, using speed as outcome, correcting for multiple participations of some athletes to the finals at World Championships or Olympic Games. Generalized mixed models with repeated measures, thereby correcting for multiple participations, were used to analyze the data and investigate a potential impact of changes in age on swimming performance. An initial mixed model was carried out, using speed as dependent variable and age, sex, country, contest (*i.e.*, World Championships *versus* Olympic Games), type of swimming strokes (*i.e.*, freestyle, breaststroke, backstroke, butterfly, and individual medley) and calendar year as independent variables.

Then, single and multi-level regression analyses were carried out for each co-variable, correcting for age of athletes in order to prevent a misinterpretation of the “age-effect” as a “time-effect” and to avoid the impact of a cluster-effect on the results regarding the analysis of age of peak swimming performance and swimming performance. To investigate whether the trend in swimming performance over time was linear or non-linear (*i.e.*, polynomial), we additionally calculated the non-linear regression model that fits the data best. The result of the linear regression analysis was compared to the best-fit result of the non-linear analysis using Akaike’s Information Criteria (AIC) and an F-test to show which model would be the most appropriate to explain the trend of the data.

Statistical analyses were performed using IBM SPSS Statistics (Version 22, IBM SPSS, Chicago, IL, USA) and GraphPad Prism (Version 6.01, GraphPad Software, La Jolla, CA, USA). Significance was accepted at *p* < 0.05 (two-sided for *t*-tests).

## 3. Results

In total, data were available for 412 performances between 1992 and 2013 with 202 women (49%) and 210 men (51%), of which 250 were competing in the World Championships and 162 in the Olympic Games. All the data (*i.e.*, age, swim speed) were characterized by normal distribution and homogeneity of variances.

### 3.1. MultiLayer Perceptron Model

**Figure 1:**
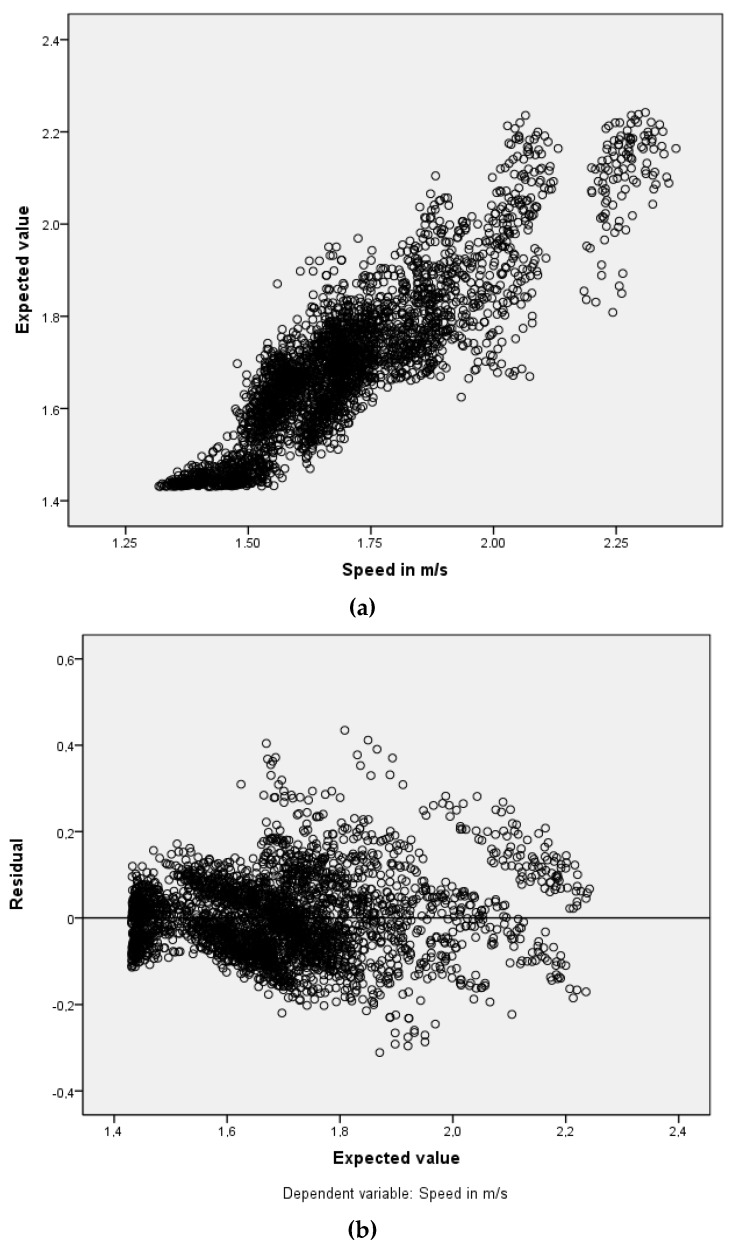
Quality of the MultiLayer Perceptron (MLP) model (observed values *versus* expected values scatterplot) (**a**) and quality of the MLP model (residual *versus* expected values scatterplot) (**b**).

MLP model proved to be a good-quality model (Figure 1). Age came out as the second variable in terms of normalized importance in predicting speed as outcome.

### 3.2. Impact of Predictor Variables on Speed

Initially, a mixed model was performed, using speed as dependent variable and all the different co-variables as independent variables, namely, sex, age, country, contest (*i.e.*, World Championship *versus* Olympic Games), type of stroke (*i.e.*, freestyle, breaststroke, backstroke, butterfly, and individual medley) and calendar year. Speed depended on all these variables but the contest (*p* = 0.211, not statistically significant) (Table 1, Table 2 and Table 3). However, in this study, we will specifically focus on the impact of age on swimming performances, while the contribution of the other co-variables will be investigated in separated contributions, currently under preparation.

### 3.3. Changes in the Ages of the Champions

**Figure 2:**
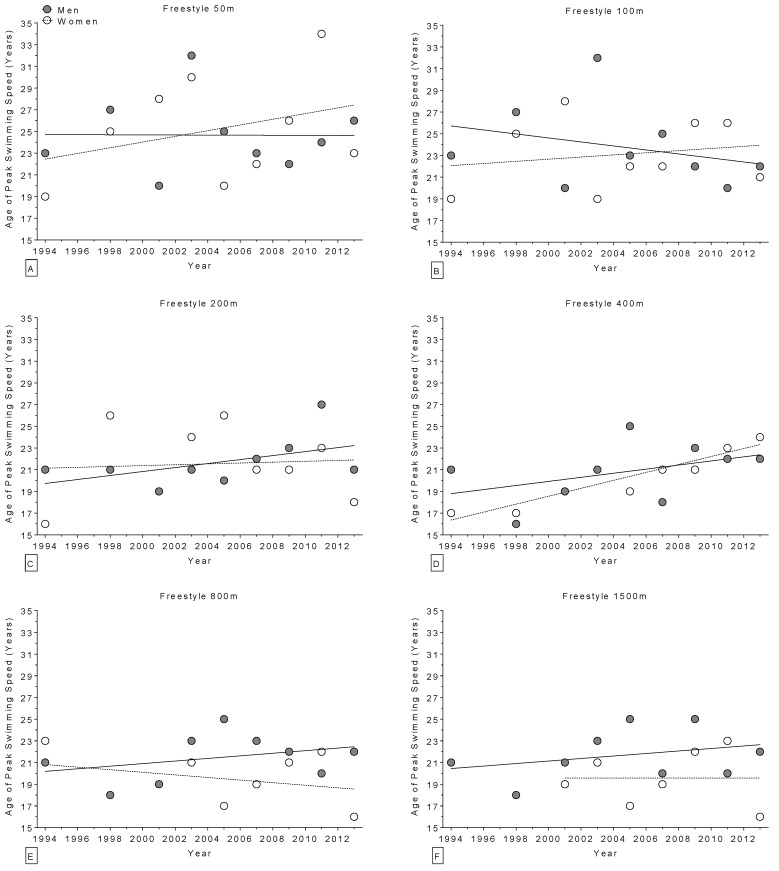
Change in the age of world champions (1994–2013) over time for freestyle 50 m (**Panel A**); 100 m (**Panel B**); 200 m (**Panel C**); 400 m (**Panel D**); 800 m (**Panel E**); and 1500 m (**Panel F**).

**Figure 3:**
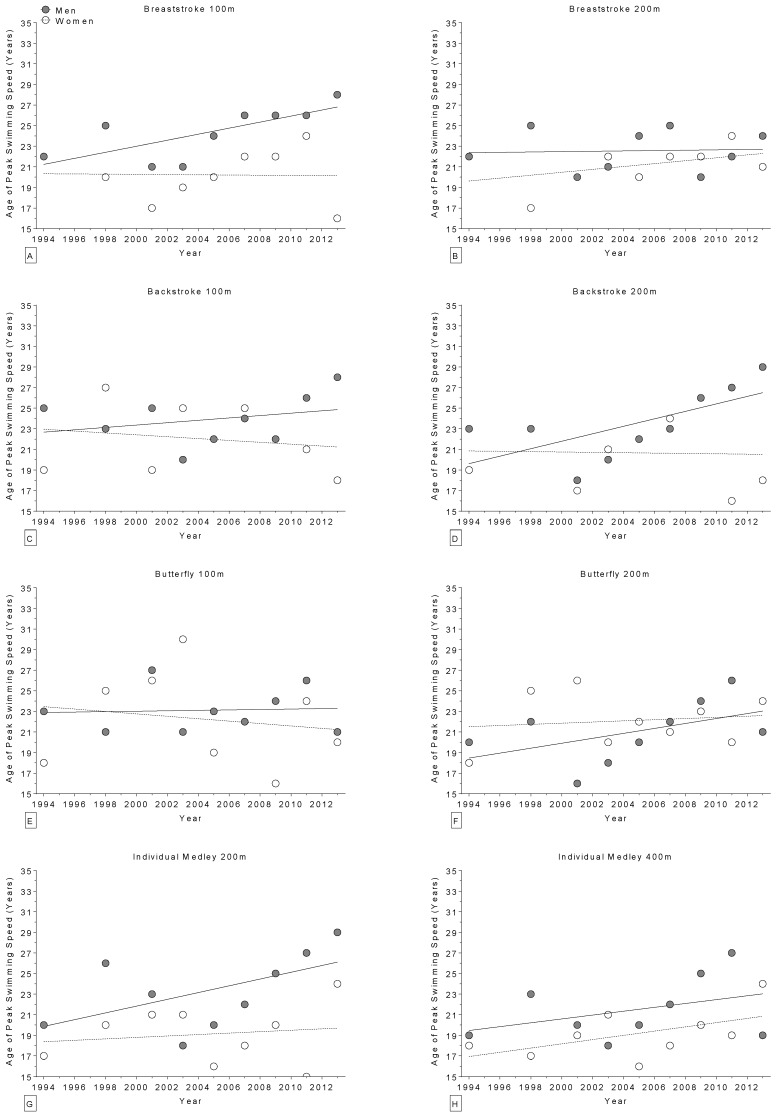
Change in the age of world champions (1994–2013) over time for 100 m breaststroke (**Panel A**); 200 m breaststroke (**Panel B**); 100 m backstroke (**Panel C**); 200 m backstroke (**Panel D**); 100 m butterfly (**Panel E**); 200 m butterfly (**Panel F**); 200 m individual medley (**Panel G**); and 400 m individual medley (**Panel H**).

**Figure 4:**
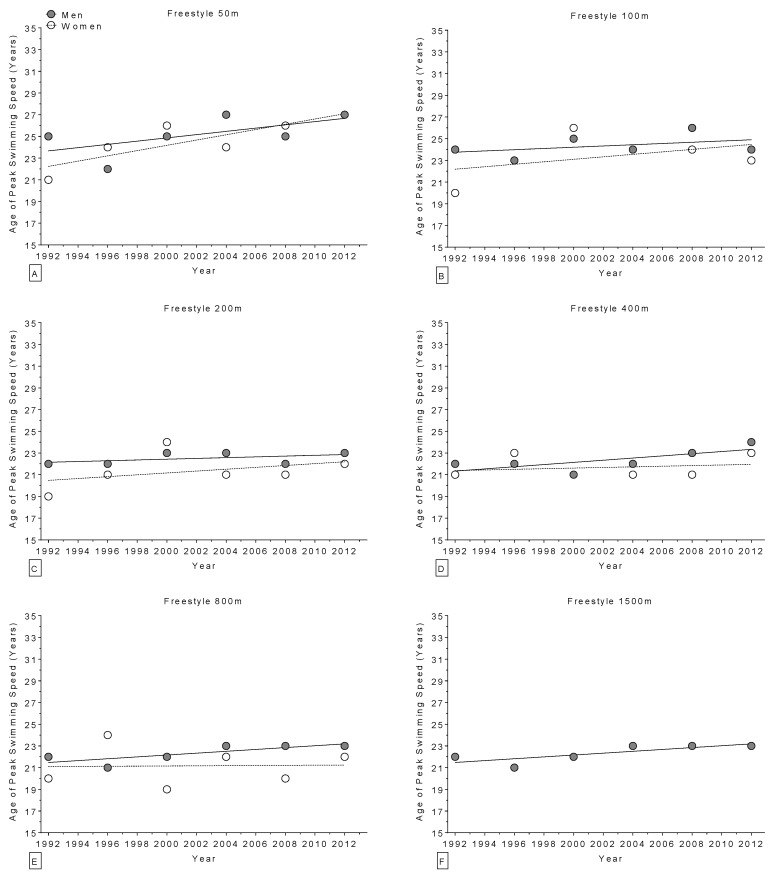
Change in the age of Olympic champions (1992–2012) over time in freestyle for 50 m (**Panel A**); 100 m (**Panel B**); 200 m (**Panel C**); 400 m (**Panel D**); 800 m (**Panel E**); and 1500 m (**Panel F**).

**Figure 5:**
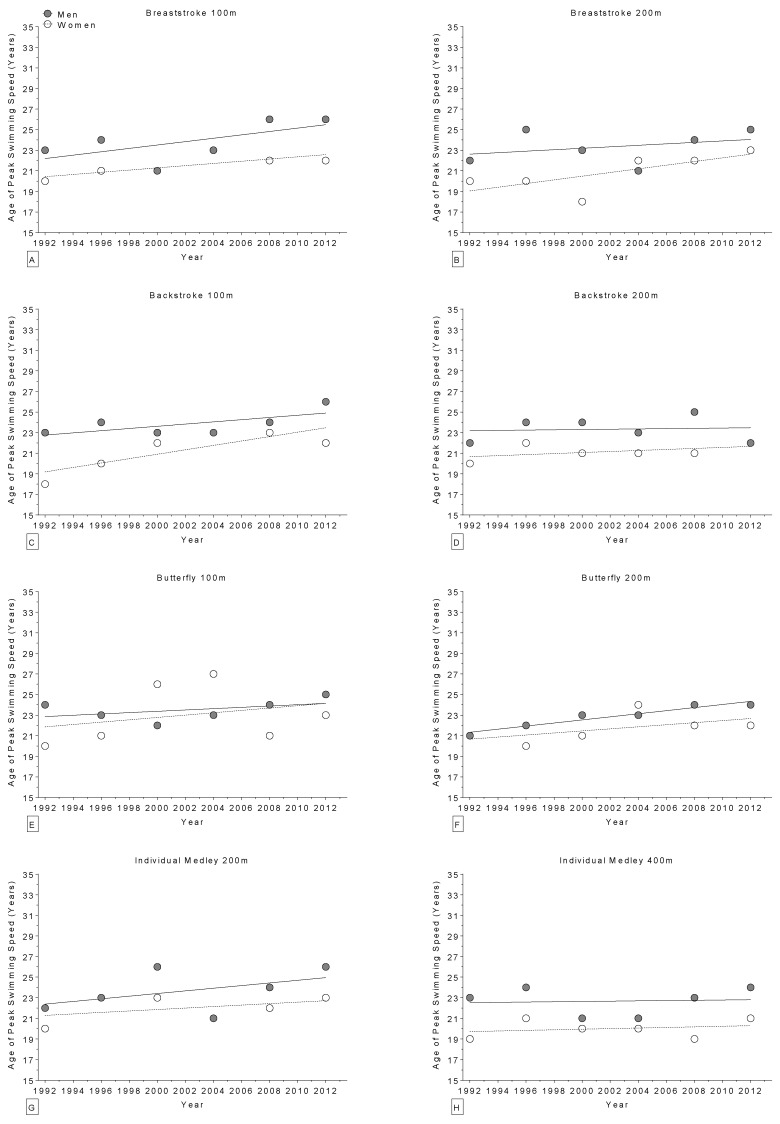
Change in the age of Olympic champions (1992–2012) over time for 100 m breaststroke (**Panel A**); 200 m breaststroke (**Panel B**); 100 m backstroke (**Panel C**); 200 m backstroke (**Panel D**); 100 m butterfly (**Panel E**); 200 m butterfly (**Panel F**); 200 m individual medley (**Panel G**); and 400 m individual medley (**Panel H**).

The age of World champions increased over the years in the 400 m freestyle for women (Figure 2) and in the 100 m breaststroke for men (Figure 3), but remained stable (Table 4) in all other disciplines (Figure 2 and Figure 3). Regarding the Olympic champions, the age remained stable (Table 5) in all disciplines (Figure 4 and Figure 5). Longer race distances (*i.e.*, 200 m and over) were completed by younger champions (~20 years for women and ~22 years for men) than shorter (*i.e.*, 50 m and 100 m) race distances (~22 years for women and ~24 years for men) for each stroke and in both women and men (Table 4 and Table 5). Overall, the mean ages of champions were 21 ± 3.5 years for women (minimum of 14 years, maximum of 34 years; 21 ± 3.4 at the World Championships and 21 ± 3.6 years at the Olympic Games) and 23 ± 2.8 years for men (minimum of 16 years, maximum of 32 years; 23 ± 3.0 years at the World Championships and 23 ± 2.5 years at the Olympic Games). The mean sex difference in the age of champions was ~2 years in both the World Championships and the Olympic Games, even if, when correcting for all the co-variables, the type of contest had no statistically significant impact on swimming performance (Table 1 and Table 2).

### 3.4. Changes in Swimming Performances across the Years

**Figure 6:**
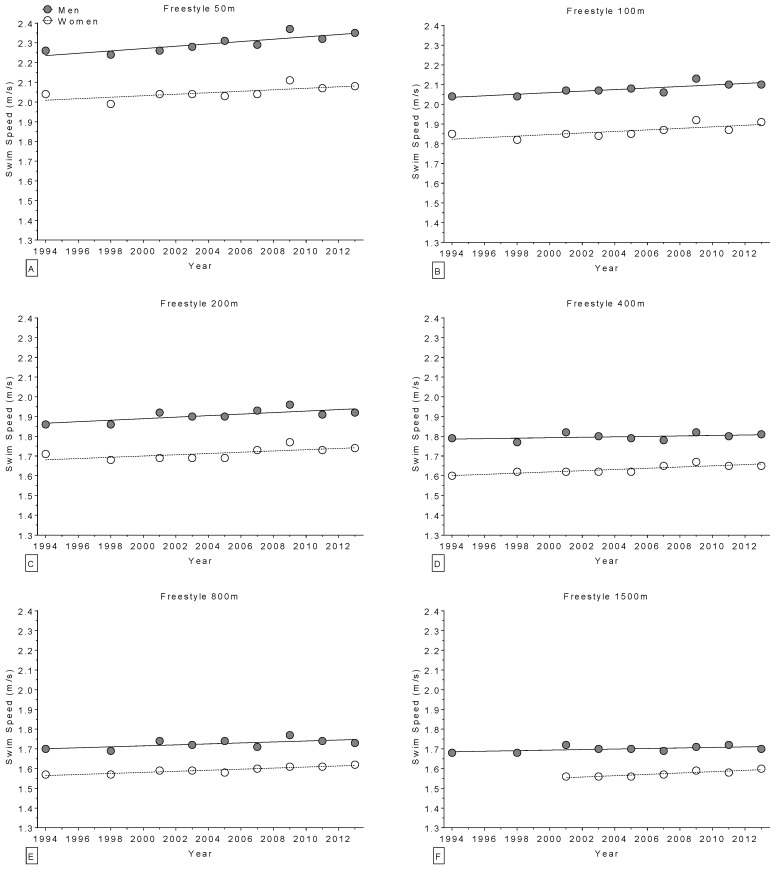
Change in freestyle swimming speed over time in world champions (1994–2013) for 50 m (**Panel A**); 100 m (**Panel B**); 200 m (**Panel C**); 400 m (**Panel D**); 800 m (**Panel E**); and 1500 m (**Panel F**).

**Figure 7:**
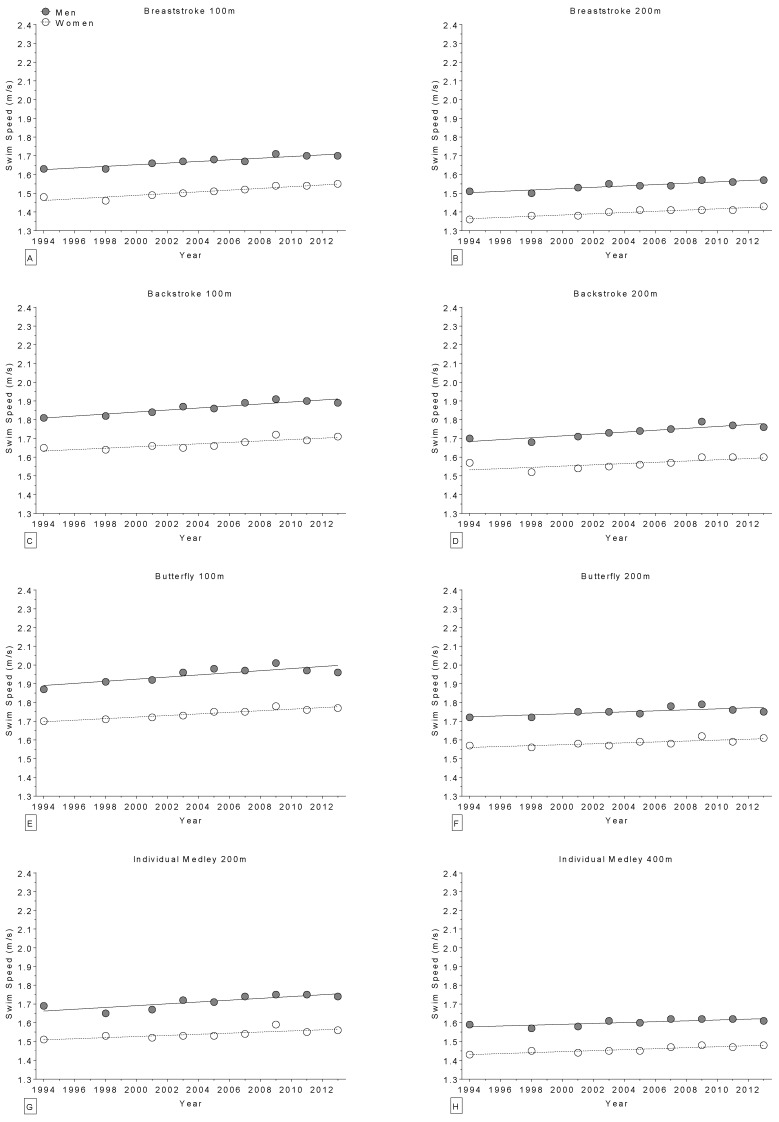
Change in swimming speed over time in world champions (1994–2013) for 100 m breaststroke (**Panel** A); 200 m breaststroke (**Panel B**); 100 m backstroke (**Panel C**); 200 m backstroke (**Panel D**); 100 m butterfly (**Panel E**); 200 m butterfly (**Panel F**); 200 m individual medley (**Panel G**); and 400 m individual medley (**Panel H**).

**Figure 8:**
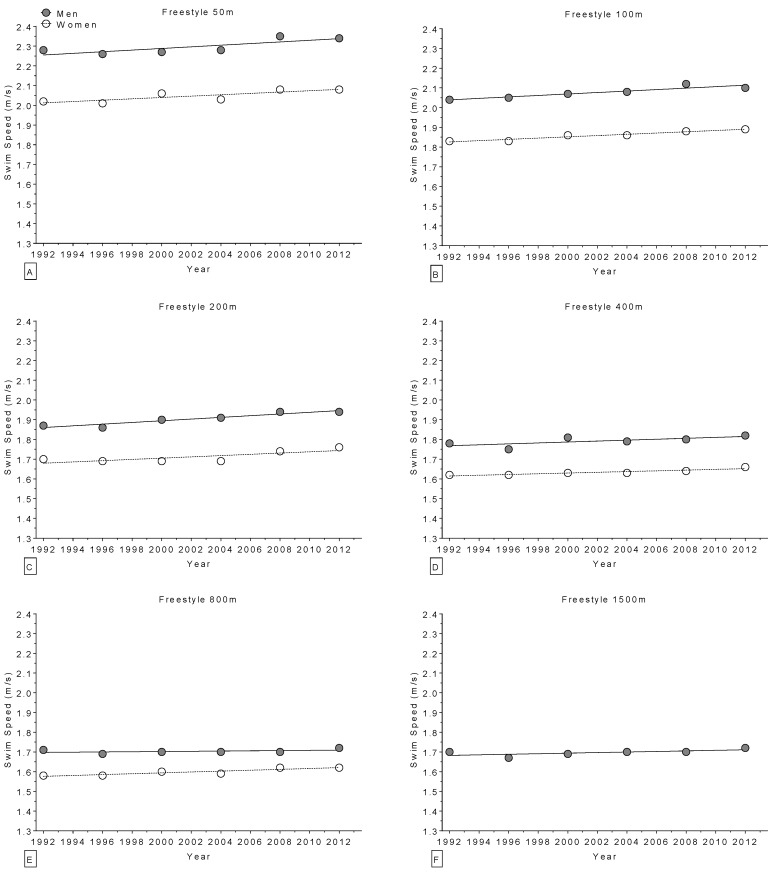
Change in freestyle swimming speed over time in Olympic champions (1992–2012) for 50 m (**Panel A**); 100 m (**Panel B**); 200 m (**Panel C**); 400 m (**Panel D**); 800 m (**Panel E**); and 1500 m (**Panel F**).

**Figure 9:**
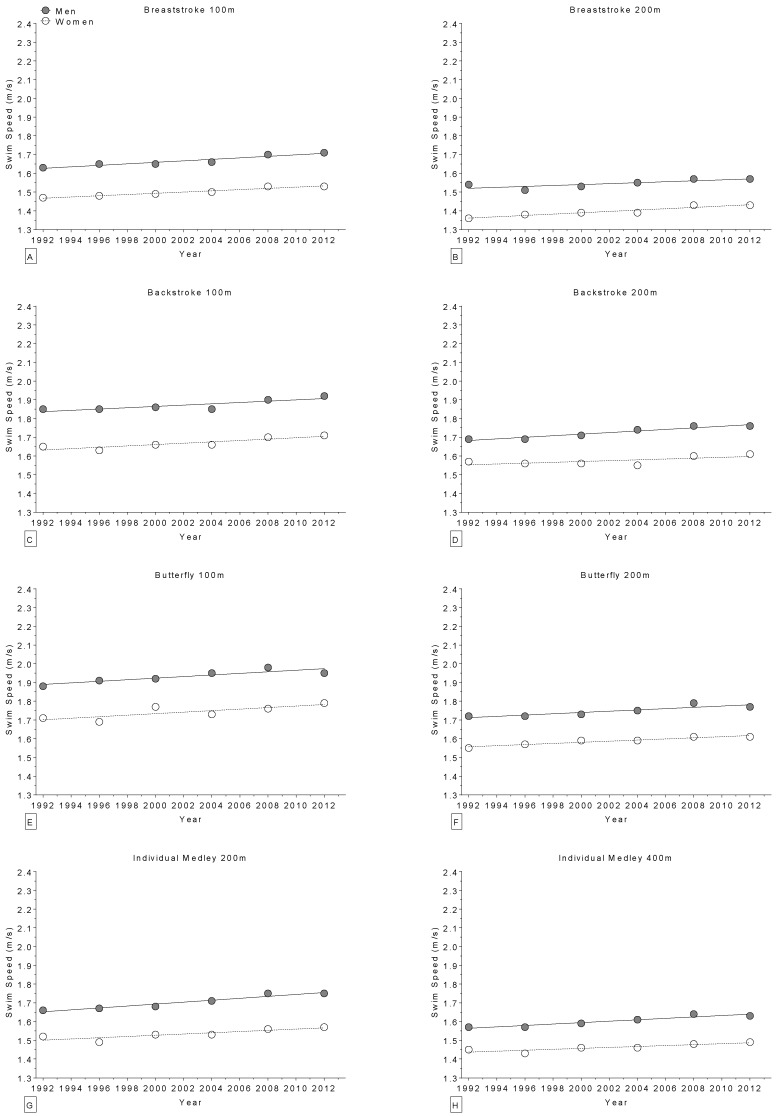
Change in swimming speed over time in Olympic champions (1992–2012) for 100 m breaststroke (**Panel A**); 200 m breaststroke (**Panel B**); 100 m backstroke (**Panel C**); 200 m backstroke (**Panel D**); 100 m butterfly (**Panel E**); 200 m butterfly (**Panel F**); 200 m individual medley (**Panel G**); and 400 m individual medley (**Panel H**).

In world champions, swimming performance improved linearly and significantly across calendar years (Table 6) in 21 of 28 distances of freestyle (Figure 6), breaststroke, backstroke, butterfly and individual medley (Figure 7), with the exception of one non-linear increase in the 200 m backstroke for women. Regarding the Olympic champions, swimming performance improved significantly across calendar years (Table 7) in 16 of 27 distances of freestyle (Figure 8), breaststroke, backstroke, butterfly and individual medley (Figure 9). All results were corrected for co-variables and remained unmodified.

## 4. Discussion

The present study examined the changes in age and swimming performance of female and male world and Olympic champions for all strokes and race distances in indoor, long course pools. The main findings were: (1) the age of champions remained stable in most race distances; (2) longer race distances (*i.e.*, 200 m and over) were completed by younger champions than shorter race distances (*i.e.*, 50 m and 100 m) for each stroke, and in both women and men; (3) female champions were ~2 years younger than male champions with a mean age of ~21 and ~23 years for women and men, respectively; (4) swimming performance improved in most race distances for world and Olympic champions, but remained stable in several race distances.

### 4.1. Unchanged Age of Peak Swimming Performance in Champions over Time

A first important finding was that the age of peak swimming performance for champions at the world class level remained stable in most strokes and race distances. The only increases in the age of world champions were found in the freestyle 400 m for women and in the breaststroke 100 m for men. Schulz and Curnow described the age of peak performance of Olympic gold medal winners as unchanged at ~18 years for women and ~21 years for men for almost a century [1]. In comparison with this, the present champions competing in the last ~20 years are ~2–3 years older, with a mean age of 21 ± 3.5 years in women and 23 ± 2.8 years in men.

Nevertheless, there are no more studies investigating the champions. However, several studies focus on top swimmers [57,58,59,60,61,62,63] for different strokes and distances. For example, Allen *et al.* focused on the top 16 pool events held at the 2008 and 2012 Olympic Games [57]. They presented a result for the age of the top 16, which is comparable to the age of champions in the present study, with a difference of approximately one year for the older swimmers in the study by Allen *et al.* [57]. Interestingly, Allen *et al.* reported that the age for the freestyle 50 m was also higher in women than in men, as is the case in the present study [57]. In all the other distances, the results were reversed, with higher ages in men compared to women [57].

Moreover, there seems to be a trend of an increase in the age of finalists over time as it was described recently for the same time period, strokes and competitions [3]. König *et al.* [3] reported in the case of the World Championships that the age of the finalists increased for all strokes and distances with the exception of the 200 m backstroke in women, and the 400 m freestyle and the 200 m breaststroke in men, where the age of the finalists decreased. In the Olympic Games, the age of the finalists increased for all strokes and distances with the exception of the 800 m freestyle in women and the 400 m individual medley in men. This finding for finalists in elite swimming competitions is in line with other sports disciplines such as running and triathlon [64,65], where the increase in the age was attended by a continuous improvement in performance. Examples were given for the “Ironman Hawaii” in the study by Gallmann *et al.*, with an increase from ~26 years to ~35 years for women and from ~27 years to ~34 years for men in the time period of 1983 to 2012 [65]. During the same period, race times of the elite athletes decreased [65].

Thus, is there any chance for a previous top swimmer like Michael Phelps to win another medal in a competition at the world class level in a comeback after his retirement? The mean age of champions is far below 30 years. Nevertheless, three male champions (*i.e.*, Aleksandr Vladimirovich Popov, 50 m freestyle at the World Championships in 2003; Roland Schoeman, 50 m freestyle at the World Championships in 2013; and Jason Lezak, 100 m freestyle at the Olympic Games in 2008) and six female champions (*i.e.*, Claudia Poll, 400 m freestyle at the World Championships in 2005; Dara Torres, 50 m freestyle, 100 m freestyle and 100 m butterfly at the Olympic Games in 2000; Marleen Veldhuis, 50 m freestyle at the Olympic Games in 2012; and Svitlana Bondarenko, 100 m breaststroke at the Olympic Games in 2004) were 33 years old [65]. There was even one female champion (Therese Alshammar) who won in 2011 at the World Championships in the 50 m freestyle at the age of 34 [66]. Aleksandr Vladimirovich Popov from Russia showed that it is possible to win a gold medal in swimming after age 30. In 1992 (at age 20) at the Barcelona Olympics, he won the 50 m freestyle with a time of 21.91 s. In 2003 (at age 31) in the same pool in Barcelona at the World Championships, he won the 50 m freestyle with a time of 21.92 s [67]. It is also worth mentioning that Dara Torres won the silver medal in 50 m freestyle at the 2008 Olympic Games in Beijing at the age of 41 [56]. Moreover, in other sports disciplines such as running there are some exceptional athletes who surprised with their outstanding performance. For example, a 90-year old marathon runner officially became the first person to finish a whole marathon at this age [68]. His official finishing time was 6 h, 48 min and 55 s. Müller *et al.* investigated the physiological alterations in that exceptional race [69]. Therefore, we assume that there is also still a certain chance to win a gold medal at the world class level for swimmers over 30 years of age and for athletes intending to make a comeback at that age.

### 4.2. Younger Swimmers Were Faster in Longer Race Distances

In accordance to other studies investigating the association of age and race distances [1,3,58,59,61,62], we also came to a second important finding that longer race distances were associated with younger swimmers’ age for all strokes and in both women and men. Tanaka and Seals assumed that this phenomenon was based on the different energy-producing pathways in skeletal muscle involved with diverse intensities [70].

This important finding could be explained through physiological and biological factors.

For sprint distances (e.g., 50 m freestyle), an athlete’s body uses an energy-pathway that is fast enough to deliver the required energy in a foreseeable time. That can be achieved by anaerobic metabolism [71,72]. Aerobic metabolism delivers the needed energy for endurance sports disciplines and endurance swimming distances (e.g., 1500 m freestyle) [73]. Furthermore, Larsson *et al.* reported that there is an increase of isometric and dynamic strength until the age of ~30 years [74]. An improvement in swimming performance until the age of ~30 years can be assumed for sprint distances. For endurance distances, a decrease in metabolic rates, longer response times, poorer coordination and a reduced maximum oxygen uptake (VO_2_ max) after the age of ~20 might explain the younger swimmers’ age [75,76,77].

### 4.3. Female Champions Were Younger than Male Champions

A third important finding was that female champions were younger than male champions with a difference of ~2 years. That is in line with other studies reporting a sex difference of ~2 years. Schulz and Curnow examined Olympic champions from 1896 to 1980 [1]. They reported a constant sex difference in the age of peak swimming performance of ~2 years. Different reasons have been assumed to explain that sex difference. König *et al.* pointed to the different maturation processes during puberty [3]. Most important parameter in this context is body height [2], growth velocity that was not reached until the age of ~12 years in girls and ~14 years in boys [78], the different onset of puberty at ~11 years in girls and ~13 years, expressed as the skeletal/biological age [79], different bone growth (*i.e.*, metacarpal thickness, width, length and medullary diameter) between girls and boys.

### 4.4. Increase of Peak Swimming Performance across the Years

In 1997, Tanaka and Seals [70] described an interaction between age and sex in freestyle swimming. They concluded that the rate of decline with advancing age appeared to be associated with the length of the performance (*i.e.*, from 50 to 1500 m) and with the sex. These female and male world and Olympic champions improved swimming performance in most race distances with a larger trend of improvement observed in Olympic champions, although the age of peak swimming performance remained stable in most race distances for World champions and in all race distances for Olympic champions. These findings differ from recent findings for finalists competing in World Championships and Olympic Games where performance improved and age increased [3]. Additionally, when freestyle swimmers competing at national level were investigated (*i.e.*, Swiss swimmers between 1994 and 2012), the age of peak swimming speed was younger in women compared to men for the 50 m to the 800 m freestyle. For women, the age of peak swimming speed increased over time in the 50 m but decreased over time in the 1500 m freestyle. For men, the age of peak swimming speed decreased over time in the 50 m freestyle [80].

As Schulz and Curnow reported, swimming performance changed “dramatically” between 1896 and 1980 [1]. They explained this enormous increase in performance by external factors such as improved training programs and diets, better equipment, larger population bases from which athletes are selected, as well as more efficient selection strategies. We can only agree with this. All those factors changed over time. The equipment (e.g., swimsuits), to a certain extent, helps swimmers reach peak swimming speed [5]. It has been shown that wearing a full-body suit or a waist-to-ankle suit significantly reduced passive drag. The reduction in drag was associated with a decreased energy cost of sub-maximal swimming and an increased distance per stroke, at the same stroke rates, and reduced freestyle performance time [81].

Different studies investigating peak swimming performance showed controversial results concerning its evolution over time [59,82]. Buhl *et al.* [59] described a limit in performance at the international level for freestyle, while they showed that there was an increase of up to 5.3% at national (*i.e.*, Switzerland) level. Koch-Ziegenbein *et al.* showed an increase in swimming performance of 1.2%–5.2% at international level for breaststroke [82]. The results in the present study are more stable concerning the swimming performance, compared to the results from the study of König *et al.* describing swimming performance in finalists [3]. This might strengthen the prediction of a limit in swimming performance. However, as König *et al.* already reported, the trend in progression of peak swimming performance remains a controversial question and needs further investigation [3].

As a result of newly introduced swimsuits [5], numerous word records were set in 2009. Some investigations showed that swimming performance would reach its limit [4,58,83]. Berthelot described that swimming performance may soon reach its limit [58]. Nevill *et al.* analyzed front-crawl swimming from 1957 to 2007 [83]. They proclaimed that a non-linear, flattened S-shaped logistic curve would fit the data best. In the present study we assumed that linear regression would describe the data best. We also might not have investigated a time period which is long enough to assess progression in swimming performance [3].

### 4.5. Strengths and Limitations of this Study and Implications for Future Research

A strength of the present study is first that all distances and all strokes of freestyle, breaststroke, backstroke, butterfly and individual medley were included. Second, the topic from this study is up to date and interesting for today’s world class swimmers. Nevertheless, the present study is limited in terms of missing variables such as anthropometric [2,84,85,86], biological [1,74,75,76], and physiological factors [67,70,83,84,87,88]. For calculating the age of champions at the particular event, the year of birth and the exact date of event were considered. It was not possible to find all the exact dates of birth for each swimmer. The website from where the data analyzed in the present study were retrieved [89] provides only the year of birth of the athletes, but the exact date of events. Thus, we accepted this lack of precision on the assumption that the differences will compensate one another by a normal distribution. Consequently further research is needed on the age of peak swimming performance, its change over the years, and its comportment in different strokes and race distances as in the present study, in recent years and also at the international level. A further limitation is that we did not consider whether or not athletes were wearing non-textile suits/full-body suits during this period [90], a factor that might influence race time through its effect on passive drag [81,91]. Furthermore, beginning with the season 2013–2014, the Fédération Internationale de Natation (FINA) changed the rules for breaststroke swimming and, therefore, the new rule changes are likely to see faster swims [92].

### 4.6. Practical Applications

In swimming, longer race distances were completed at younger ages than shorter race distances. The opposite is true for running where older athletes were faster in longer races [93]. The knowledge about the changes in the age of champions over time could be of great interest for swimmers competing at the world class level, as well as for their coaches and sports managers.

## 5. Conclusions

These findings suggest, first, that female (~21 years) swimming champions were ~2 years younger compared to male (~23 years) champions for all strokes and distances competing at the world class level. Second, longer (200 m and over) race distances were completed by younger champions (~20–22 years) compared to shorter (50 m and 100 m) race distances (~22–24 years). Third, the age of champions remained stable over time in most strokes and distances. Fourth, swimming performance improved in all strokes and distances.

## Figures and Tables

**Table 1:** Importance of independent variables from the Multi-Layer Perceptron Analysis, using speed as outcome and correcting for multiple participations of athletes to finals of World Championship and Olympic Games.

Variable	Importance	Normalized Importance
Contest (*i.e.*, World Championship *versus* Olympic Games)	0.001	0.5%
Stroke (*i.e.*, freestyle, breaststroke, backstroke, butterfly, and individual medley)	0.265	100.0%
Sex	0.109	41.1%
Country	0.168	63.5%
Year	0.023	8.8%
Age	0.190	71.5%

**Table 2:** Fixed effects of the mixed model with speed as dependent variable and age, sex, country, calendar year, contest (*i.e.*, World Championships *versus* Olympic Games) and stroke (*i.e.*, freestyle, breaststroke, backstroke, butterfly, and individual medley) as independent variables.

Parameters	*F*-Statistics	*p*-Value
Intercept	23.958	<0.0001
World Championship *versus* Olympic Games	1.564	0.211
Contest	603.892	<0.0001
Sex	937.596	<0.0001
Country	7.685	<0.0001
Year	46.921	<0.0001
Age	167.531	<0.0001

**Table 3:** Estimates of the mixed model with speed as dependent variable and age, sex, country, calendar year, contest (*i.e.*, World Championship *versus* Olympic Games) and stroke (*i.e.*, freestyle, breaststroke, backstroke, butterfly, and individual medley) as independent variables.

Parameter	Estimate	SE	*T*	*p*	95% Confidence Interval	
					Lower Limit	Upper Limit
Intercept	−3.682	0.741	−4.967	<0.0001	−5.135	−2.229
World Championship	−0.006	0.005	−1.251	0.211	−0.015	0.003
Freestyle	0.245	0.007	35.561	<0.0001	0.232	0.259
Breaststroke	−0.047	0.008	−5.643	<0.0001	−0.063	−0.030
Backstroke	0.138	0.008	16.670	<0.0001	0.122	0.154
Butterfly	0.163	0.008	19.601	<0.0001	0.146	0.179
Females	−0.147	0.005	−30.620	<0.0001	−0.156	−0.137
(Country = ALG)	0.252	0.077	3.266	0.001	0.101	0.403
(Country = ARG)	−0.038	0.062	−0.616	0.538	−0.161	0.084
(Country = AUS)	0.021	0.030	0.700	0.484	−0.037	0.078
(Country = AUT)	0.011	0.037	0.284	0.777	−0.063	0.084
(Country = BAH)	0.300	0.092	3.255	0.001	0.119	0.481
(Country = BAR)	0.218	0.127	1.715	0.086	−0.031	0.467
(Country = BEL)	−0.014	0.046	−0.311	0.756	−0.105	0.076
(Country = BLR)	0.127	0.041	3.091	0.002	0.046	0.207
(Country = BRA)	0.140	0.034	4.164	<0.0001	0.074	0.206
(Country = BUL)	0.069	0.127	0.542	0.588	−0.181	0.318
(Country = CAN)	−0.014	0.031	−0.451	0.652	−0.075	0.047
(Country = CHI)	−0.196	0.068	−2.876	0.004	−0.330	−0.062
(Country = CHN)	0.023	0.030	0.758	0.449	−0.037	0.083
(Country = CRC)	−0.093	0.045	−2.078	0.038	−0.182	−0.005
(Country = CRO)	0.134	0.046	2.912	0.004	0.044	0.224
(Country = CUB)	0.060	0.053	1.136	0.256	−0.043	0.163
(Country = CZE)	−0.031	0.050	−0.625	0.532	−0.130	0.067
(Country = DEN)	−0.011	0.034	−0.322	0.747	−0.077	0.055
(Country = ESP)	−0.044	0.034	−1.288	0.198	−0.110	0.023
(Country = EST)	0.232	0.127	1.825	0.068	−0.017	0.482
(Country = EUN)	0.078	0.039	1.981	0.048	0.001	0.154
(Country = FAR)	−0.180	0.069	−2.633	0.009	−0.315	−0.046
(Country = FIN)	0.053	0.046	1.153	0.249	−0.037	0.143
(Country = FRA)	0.043	0.031	1.394	0.163	−0.018	0.104
(Country = GBR)	−0.027	0.030	−0.887	0.375	−0.086	0.032
(Country = GER)	−0.004	0.030	−0.142	0.887	−0.063	0.055
(Country = GRE)	−0.047	0.042	−1.113	0.266	−0.130	0.036
(Country = HUN)	0.008	0.031	0.261	0.794	−0.052	0.068
(Country = IRL)	−0.078	0.058	−1.340	0.180	−0.192	0.036
(Country = ISL)	0.017	0.077	0.226	0.821	−0.134	0.169
(Country = ISR)	0.062	0.055	1.125	0.261	−0.046	0.170
(Country = ITA)	−0.036	0.031	−1.167	0.243	−0.097	0.025
(Country = JAM)	−0.009	0.092	−0.093	0.926	−0.190	0.172
(Country = JPN)	−0.003	0.030	−0.090	0.928	−0.061	0.056
(Country = KAZ)	−0.005	0.077	−0.067	0.947	−0.157	0.147
(Country = KEN)	0.147	0.069	2.135	0.033	0.012	0.281
(Country = KOR)	−0.044	0.045	−0.979	0.328	−0.133	0.044
(Country = LTU)	0.100	0.046	2.164	0.031	0.009	0.190
(Country = MAS)	0.063	0.127	0.497	0.619	−0.186	0.312
(Country = MDA)	−0.051	0.092	−0.551	0.582	−0.232	0.130
(Country = NED)	0.115	0.032	3.625	<0.0001	0.053	0.177
(Country = NOR)	−0.015	0.049	−0.302	0.763	−0.110	0.081
(Country = NZL)	−0.058	0.037	−1.555	0.120	−0.131	0.015
(Country = PNG)	0.100	0.127	0.783	0.434	−0.150	0.349
(Country = POL)	−0.004	0.033	−0.110	0.912	−0.068	0.061
(Country = PUR)	0.176	0.059	3.020	0.003	0.062	0.291
(Country = ROU)	−0.066	0.034	−1.957	0.050	−0.132	0.000
(Country = RSA)	0.071	0.033	2.187	0.029	0.007	0.135
(Country = RUS)	0.031	0.031	1.011	0.312	−0.029	0.091
(Country = SIN)	0.124	0.127	0.975	0.330	−0.125	0.373
(Country = SLO)	−0.043	0.047	−0.912	0.362	−0.135	0.050
(Country = SRB)	0.065	0.055	1.176	0.240	−0.043	0.173
(Country = SUI)	−0.091	0.049	−1.872	0.061	−0.187	0.004
(Country = SUR)	0.079	0.092	0.855	0.393	−0.102	0.260
(Country = SVK)	0.073	0.041	1.790	0.074	−0.007	0.154
(Country = SWE)	0.133	0.033	3.986	<0.0001	0.068	0.199
(Country = TCH)	−0.088	0.127	−0.693	0.488	−0.338	0.161
(Country = TPE)	0.006	0.127	0.050	0.960	−0.243	0.256
(Country = TRI)	0.250	0.055	4.547	<0.0001	0.142	0.358
(Country = TUN)	−0.129	0.041	−3.168	0.002	−0.208	−0.049
(Country = UKR)	0.019	0.033	0.585	0.559	−0.045	0.084
(Country = USA)	0.033	0.029	1.142	0.254	−0.024	0.091
(Country = VEN)	0.072	0.055	1.301	0.193	−0.036	0.180
(Country = YUG)	0.194	0.127	1.522	0.128	−0.056	0.443
(Country = ZIM) *reference*	–	–	–	–	–	–
Year	0.003	0.000	6.850	<0.0001	0.002	0.003
Age	0.009	0.002	12.943	<0.0001	0.008	0.010

**Table 4:** Changes in the age (years) of World champions over time.

Stroke and Distance	Sex	1994	Age	2013
Freestyle				
50 m	Women	†	24.0 ± 2.1	†
	Men	†	22.6 ± 1.5	†
100 m	Women	†	22.8 ± 1.3	†
	Men	†	24.2 ± 1.0	†
200 m	Women	†	21.7 ± 1.2	†
	Men	†	22. ± 0.7	†
400 m	Women	21.6 ± 0.9	–	21.6 ± 0.9
	Men	†	22.3 ± 1.4	†
800 m	Women	†	20.9 ± 1.1	†
	Men	†	21.8 ± 3.1	†
1500 m	Women	†	21.8 ± 4.7	†
	Men	†	21.9 ± 1.1	†
Breaststroke				
100 m	Women	†	21.3 ± 1.6	†
	Men	23.0 ± 2.2	–	25.3 ± 3.8
200 m	Women	†	21.8 ± 4.7	†
	Men	†	22.9 ± 0.3	†
Backstroke				
100 m	Women	†	21.4 ± 0.9	†
	Men	†	23.2 ± 1.2	†
200 m	Women	†	20.3 ± 1.2	†
	Men	†	23.2 ± 1.0	†
Butterfly				
100 m	Women	†	22.2 ± 1.2	†
	Men	†	22.5 ± 2.6	†
200 m	Women	†	20.0 ± 2.7	†
	Men	†	21.0 ± 2.3	†
Individual Medley				
200 m	Women	†	21.2 ± 0.8	†
	Men	†	23.7 ± 1.5	†
400 m	Women	†	20.4 ± 1.0	†
	Men	†	22.1 ± 0.9	†

Note: † = no change between 1992 and 2013.

**Table 5:** Changes in the age (years) of Olympic champions over time.

Stroke and Distance	Sex	1992	Age	2012
Freestyle				
50 m	Women	†	26.9 ± 5.1	†
	Men	†	27.1 ± 3.6	†
100 m	Women	†	23.3 ± 2.0	†
	Men	†	24.3 ± 1.0	†
200 m	Women	†	21.3 ± 1.6	†
	Men	†	22.5 ± 0.5	†
400 m	Women	†	21.7 ± 1.0	†
	Men	†	22.3 ± 1.0	†
800 m	Women	†	21.2 ± 1.8	†
	Men	†	22.3 ± 0.8	†
1500 m	Women	–	–	–
	Men	†	22.3 ± 0.8	†
Breaststroke				
100 m	Women	†	21.5 ± 1.0	†
	Men	†	26.4 ± 3.3	†
200 m	Women	†	20.3 ± 4.5	†
	Men	†	23.3 ± 1.6	†
Backstroke				
100 m	Women	†	18.0 ± 2.3	†
	Men	†	23.8 ± 1.2	†
200 m	Women	†	21.2 ± 0.8	†
	Men	†	23.3 ± 1.2	†
Butterfly				
100 m	Women	†	23.0 ± 2.9	†
	Men	†	23.5 ± 1.0	†
200 m	Women	†	21.7 ± 1.4	†
	Men	†	21.3 ± 3.2	†
Individual Medley				
200 m	Women	†	22.0 ± 1.3	†
	Men	†	23.7 ± 2.1	†
400 m	Women	†	20.0 ± 0.9	†
	Men	†	22.7 ± 1.4	†

Note: † = no change between 1992 and 2012.

**Table 6:** Multi-level regression analyses for change in swimming performance of world champions using speed as dependent variable (values broken down for stroke and sex) and correcting for multiple participations and the other co-variables.

Stroke and Distance	Sex	*B*	SE (*B*)	Stand. *ß*	*T*	*p*
Freestyle						
50 m	Women	0.004	0.002	0.693	2.206	0.070
	Men	0.006	0.001	0.846	3.997	0.007
100 m	Women	0.004	0.001	0.755	2.747	0.033
	Men	0.004	0.001	0.768	3.293	0.017
200 m	Women	0.003	0.001	0.694	2.844	0.029
	Men	0.004	0.002	0.846	2.797	0.031
400 m	Women	0.005	0.002	1.282	2.335	0.058
	Men	0.001	0.001	0.259	0.672	0.527
800 m	Women	0.003	0.000	0.985	7.017	<0.001
	Men	0.002	0.001	0.593	1.754	0.130
1500 m	Women	0.003	0.001	0.906	4.285	0.013
	Men	0.001	0.001	0.514	1.478	0.190
Breaststroke						
100 m	Women	0.005	0.001	0.943	7.108	<0.001
	Men	0.005	0.001	1.126	6.187	0.001
200 m	Women	0.004	0.000	1.022	8.260	<0.001
	Men	0.004	0.000	0.926	9.188	<0.001
Backstroke						
100 m	Women	0.004	0.001	0.800	3.970	0.007
	Men	0.006	0.001	1.003	8.373	<0.001
200 m	Women	0.003	0.001	0.730	2.646	0.038
	Men	0.005	0.001	0.854	3.346	0.016
Butterfly						
100 m	Women	0.004	0.000	0.905	9.760	<0.001
	Men	0.006	0.002	0.827	3.590	0.011
200 m	Women	0.002	0.001	0.769	3.002	0.024
	Men	0.003	0.001	0.687	2.049	0.086
Individual medley						
200 m	Women	0.003	0.001	0.754	2.930	0.026
	Men	0.006	0.001	1.037	4.527	0.004
400 m	Women	0.003	0.001	0.902	4.589	0.004
	Men	0.002	0.001	0.761	2.697	0.036

**Table 7:** Multi-level regression analyses for change in swimming performance of the Olympic champions using speed as dependent variable (values broken down for stroke and sex) and correcting for multiple participations and the other co-variables

Stroke and Distance	Sex	*B*	SE (*B*)	Stand. *ß*	*T*	*p*
Freestyle						
50 m	Women	0.004	0.001	0.884	2.822	0.067
	Men	0.004	0.001	0.826	2.862	0.064
100 m	Women	0.003	0.000	0.914	8.164	0.004
	Men	0.004	0.001	0.909	3.496	0.040
200 m	Women	0.003	0.001	0.737	2.468	0.090
	Men	0.004	0.001	0.923	4.636	0.019
400 m	Women	0.002	0.000	1.010	4.756	0.018
	Men	0.003	0.001	0.847	2.710	0.073
800 m	Women	0.002	0.001	0.891	3.354	0.044
	Men	0.001	0.001	0.483	1.083	0.358
1500 m	Women	–	–	–	–	–
	Men	0.002	0.001	0.898	2.561	0.083
Breaststroke						
100 m	Women	0.003	0.000	1.003	8.998	0.003
	Men	0.003	0.001	0.758	5.260	0.013
200 m	Women	0.004	0.001	1.053	3.885	0.030
	Men	0.003	0.001	0.798	2.410	0.095
Backstroke						
100 m	Women	0.003	0.001	0.839	2.966	0.059
	Men	0.003	0.001	0.769	4.159	0.025
200 m	Women	0.002	0.001	0.684	1.604	0.207
	Men	0.004	0.001	0.961	6.540	0.007
Butterfly						
100 m	Women	0.004	0.002	0.778	2.057	0.132
	Men	0.004	0.001	0.844	4.513	0.020
200 m	Women	0.003	0.001	0.958	5.700	0.011
	Men	0.004	0.001	1.030	3.669	0.035
Individual medley						
200 m	Women	0.002	0.001	0.500	2.025	0.136
	Men	0.005	0.001	0.956	6.424	0.008
400 m	Women	0.002	0.000	0.696	6.724	0.007
	Men	0.004	0.001	0.962	5.072	0.015
